# Supplementary material for: Genome-wide diversity in temporal and regional populations of the betabaculovirus Erinnyis ello granulovirus (ErelGV)
Source: BMC Genomics. 2018 Sep 24;19:698. doi: 10.1186/s12864-018-5070-6 (PMC6154946; doi:10.1186/s12864-018-5070-6)
Supplement: Supplementary file 1 — Showing a list of drs (direct repeats) observed in isolates of ErelGV. CNVs stand for the total of ‘Copy Number Variants’ found for each repetitive region. Values assigned to each dr correspond to the number of repeat units. (PDF 1385 kb) [file 12864_2018_5070_MOESM1_ESM.pdf]

Additional File 6. List of drs (direct repeats) observed in isolates of ErelGV. CNVs stand for the total of 'Copy Number Variants' found for each repetitive region. Values assigned to each dr correspond to the number of repeat units.

Tandem Repeat	CNVs	ErelGV-86	ErelGV-94	ErelGV-98	ErelGV-99	ErelGV-00	ErelGV-AC	ErelGV-PA
dr1	1	2 x 20 bp	2 x 20 bp	2 x 20 bp	2 x 20 bp	2 x 20 bp	2 x 20 bp	2 x 20 bp
dr2	2	2 x 46 bp	1 x 46 bp	1 x 46 bp	2 x 46 bp	2 x 46 bp	2 x 46 bp	2 x 46 bp
dr3	2	2 x 26 bp	2 x 26 bp	2 x 26 bp	2 x 26 bp	2 x 26 bp	2 x 26 bp	1 x 26 bp
dr4	1	3 x 27 bp	3 x 27 bp	3 x 27 bp	3 x 27 bp	3 x 27 bp	3 x 27 bp	3 x 27 bp
dr5	3	5 x 29 bp	5 x 29 bp	3 x 29 bp	5 x 29 bp	5 x 29 bp	3 x 29 bp	2 x 29 bp
dr6	1	3 x 25 bp	3 x 25 bp	3 x 25 bp	3 x 25 bp	3 x 25 bp	3 x 25 bp	3 x 25 bp
dr7	2	2 x 15 bp	2 x 15 bp	2 x 15 bp	2 x 15 bp	2 x 15 bp	2 x 15 bp	1 x 15 bp
dr8	3	12 x 3 bp	12 x 3 bp	12 x 3 bp	12 x 3 bp	12 x 3 bp	8 x 3 bp	7 x 3 bp
dr9	1	2 x 36 bp	2 x 36 bp	2 x 36 bp	2 x 36 bp	2 x 36 bp	2 x 36 bp	2 x 36 bp
dr10	3	8 x 24 bp	8 x 24 bp	8 x 24 bp	7 x 24 bp	7 x 24 bp	8 x 24 bp	6 x 24 bp
dr11	1	4 x 6 bp	4 x 6 bp	4 x 6 bp	4 x 6 bp	4 x 6 bp	4 x 6 bp	4 x 6 bp
dr12	1	3 x 52 bp	3 x 52 bp	3 x 52 bp	3 x 52 bp	3 x 52 bp	3 x 52 bp	3 x 52 bp
dr13	1	2 x 33 bp	2 x 33 bp	2 x 33 bp	2 x 33 bp	2 x 33 bp	2 x 33 bp	2 x 33 bp
dr14	1	2 x 18 bp	2 x 18 bp	2 x 18 bp	2 x 18 bp	2 x 18 bp	2 x 18 bp	2 x 18 bp
dr15	2	2 x 36 bp	2 x 36 bp	1 x 36 bp	2 x 36 bp	2 x 36 bp	1 x 36 bp	-
dr16	3	2 x 15 bp	2 x 15 bp	4 x 15 bp	2 x 15 bp	2 x 15 bp	4 x 15 bp	3 x 15 bp
