# Supplementary material for: Genome-wide diversity in temporal and regional populations of the betabaculovirus Erinnyis ello granulovirus (ErelGV)
Source: BMC Genomics. 2018 Sep 24;19:698. doi: 10.1186/s12864-018-5070-6 (PMC6154946; doi:10.1186/s12864-018-5070-6)
Supplement: Supplementary file 2 — Showing statistics of the sequencing libraries generated in this study. (PDF 1377 kb) [file 12864_2018_5070_MOESM2_ESM.pdf]

**Additional File 7.** Statistics of the sequencing libraries generated in this study.

| Isolate | # of reads | Mean ± SD | # of bases | Genome length (bp) | Coverage (X) |
|---|---|---|---|---|---|
| ErelGV-86 | 25564 | 283.23 ± 132.99 | 7,240,602 | 102,759 | 70.46 |
| ErelGV-94 | 22876 | 286.48 ± 131.23 | 6,553,573 | 102,726 | 63.80 |
| ErelGV-98 | 33383 | 384.4 ± 139.82 | 12,832,457 | 102,685 | 124.97 |
| ErelGV-99 | 51934 | 381.2 ± 141.51 | 19,796,984 | 102,764 | 192.65 |
| ErelGV-00 | 40083 | 280.63 ± 131.93 | 11,248,308 | 102,745 | 109.48 |
| ErelGV-AC | 28761 | 384.01 ± 139.4 | 11,044,611 | 102,741 | 107.50 |
| ErelGV-PA | 11272 | 580.71 ± 199.63 | 6,545,762 | 102,616 | 63.79 |
