# Supplementary material for: Genome-wide diversity in temporal and regional populations of the betabaculovirus Erinnyis ello granulovirus (ErelGV)
Source: BMC Genomics. 2018 Sep 24;19:698. doi: 10.1186/s12864-018-5070-6 (PMC6154946; doi:10.1186/s12864-018-5070-6)
Supplement: Supplementary file 3 — Showing the crrelations between non-synonymous substitutions and gene lengths. In these plots, each dot represents a gene depicted in the Fig. 3 of the main manuscript. A) Intra-isolate diversity of ErelGV-86 genes. B) ErelGV-94. C) ErelGV-98. D) ErelGV-99. E) ErelGV-00. F) ErelGV-AC. G) ErelGV-PA. As shown, the number of NSS per base pair (× 10− 3) and gene length (bp) have low to moderate correlation. The grey area corresponds to the 95% confidence interval, with highly conserved/diverse genes shown as outliers. (PDF 612 kb) [file 12864_2018_5070_MOESM3_ESM.pdf]

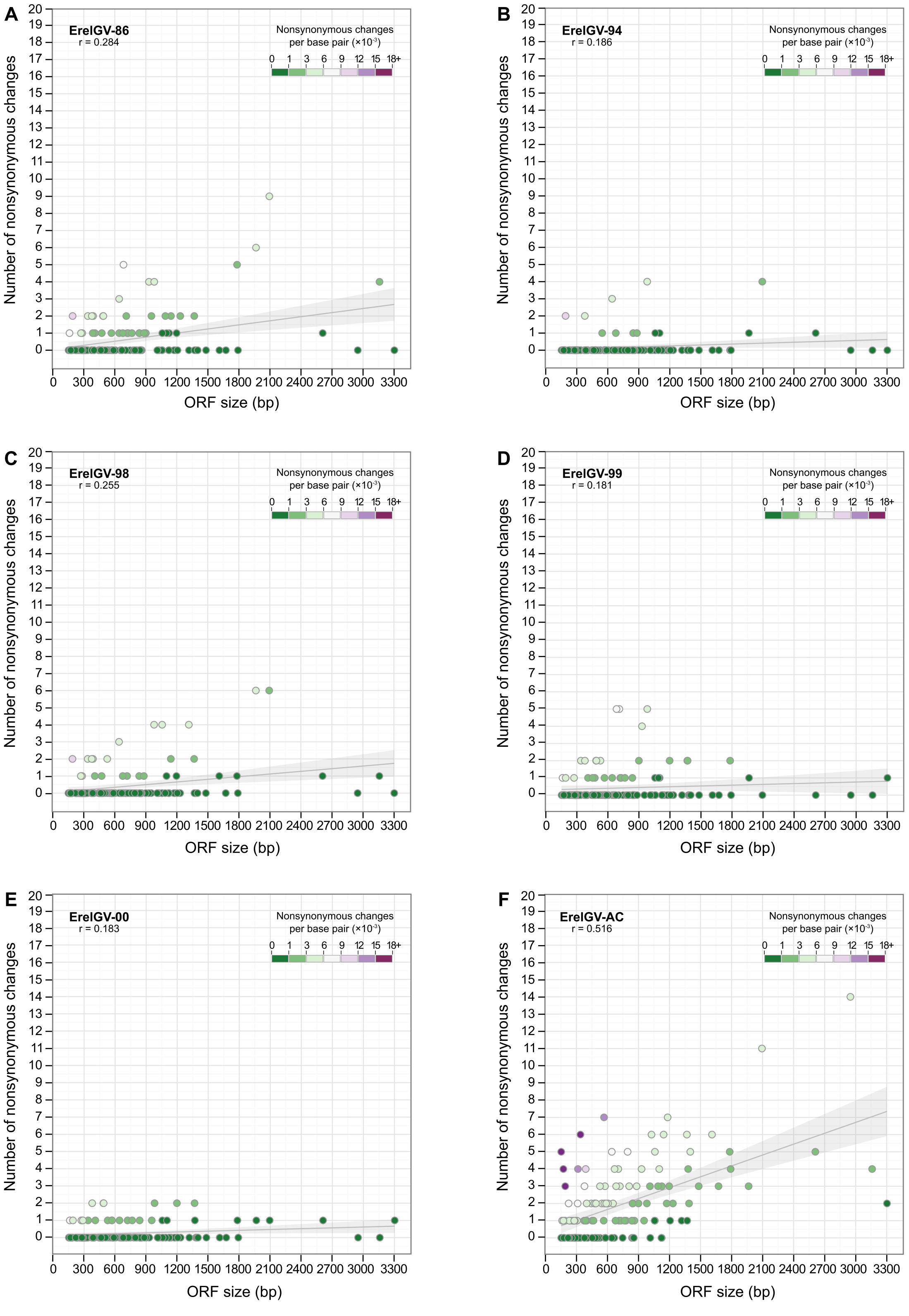

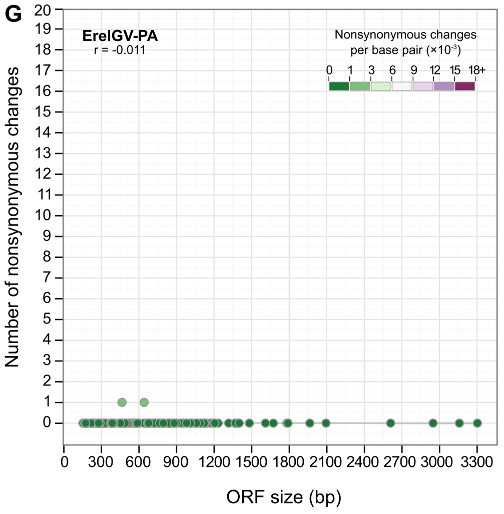

Additional file 1. Correlations between non-synonymous substitutions and gene lengths. In these plots, each dot represents a gene depicted in the Figure 3 of the main manuscript. A) Intra-isolate diversity of ErelGV-86 genes. B) ErelGV-94. C) ErelGV-98. D) ErelGV-99. E) ErelGV-00. F) ErelGV-AC. G) ErelGV-PA. As shown, the number of NSS per base pair ($\times10^{-3}$) and gene length (bp) have low to moderate correlation. The grey area corresponds to the 95% confidence interval, with highly conserved/diverse genes shown as outliers.
