# Supplementary material for: Genome-wide diversity in temporal and regional populations of the betabaculovirus Erinnyis ello granulovirus (ErelGV)
Source: BMC Genomics. 2018 Sep 24;19:698. doi: 10.1186/s12864-018-5070-6 (PMC6154946; doi:10.1186/s12864-018-5070-6)
Supplement: Supplementary file 5 — Showing a maximum likelihood tree of South American ErelGV isolates from Brazil (red dots) and Colombia (ErelGV-M34) inferred using a concatenated alignment of partial sequences of granulin, lef-9 and lef-8. (PDF 1358 kb) [file 12864_2018_5070_MOESM5_ESM.pdf]

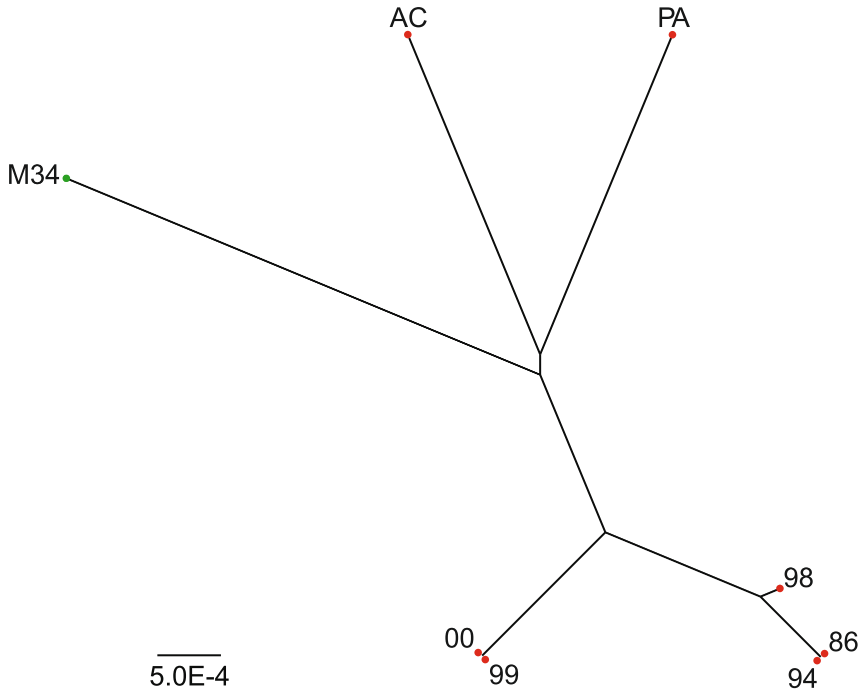

Additional File 3. Maximum likelihood tree of South American ErelGV isolates from Brazil (red dots) and Colombia (ErelGV-M34) inferred using a concatenated alignment of partial sequences of *granulin, lef-9 and lef-8*.
