# Supplementary material for: Genome-wide diversity in temporal and regional populations of the betabaculovirus Erinnyis ello granulovirus (ErelGV)
Source: BMC Genomics. 2018 Sep 24;19:698. doi: 10.1186/s12864-018-5070-6 (PMC6154946; doi:10.1186/s12864-018-5070-6)
Supplement: Supplementary file 6 — Showing the three paralogs (fgf-1, − 2, and − 3) encoded by ErelGV isolates. As observed, fgfs have different lengths (here shown in bp), and most of their polymorphisms are located in the C-terminal, and not in the region encoding the FGF domain. Their identities are low (20–33%), and mostly restricted to their central regions, responsible by encoding their main functional domain. Additional studies would be relevant to understand the roles of such proteins on ErelGV infection. (PDF 1408 kb) [file 12864_2018_5070_MOESM6_ESM.pdf]

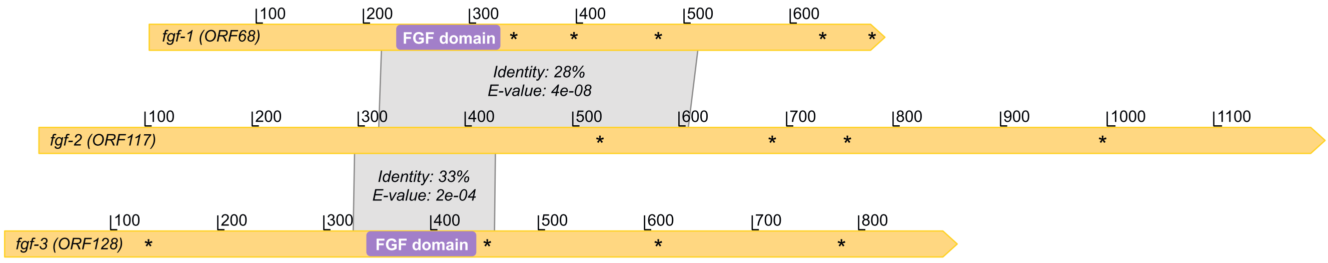


**Additional File 4.** The three paralogs (*fgf-1, -2*, and *-3*) encoded by ErelGV isolates. As observed, fgfs have different lengths (here shown in bp), and most of their polymorphisms are located in the C-terminal, and not in the region encoding the FGF domain. Their identities are low (20-33%), and mostly restricted to their central regions, responsible by encoding their main functional domain. Additional studies would be relevant to understand the roles of such proteins on ErelGV infection.
