# Supplementary material for: Genome-wide diversity in temporal and regional populations of the betabaculovirus Erinnyis ello granulovirus (ErelGV)
Source: BMC Genomics. 2018 Sep 24;19:698. doi: 10.1186/s12864-018-5070-6 (PMC6154946; doi:10.1186/s12864-018-5070-6)
Supplement: Supplementary file 7 — Showing polymorphisms in lef genes. Non-synonymous mutations in lef-1 (A) and lef-2 (B) were mainly found on the C-terminal of the proteins they encode. For LEF-10, polymorphisms were found mainly on it N-terminal (C). The exception is a single non-synonymous substitution at a region overlapping with vp1054, which in turn causes a synonymous change (CTG → CTA) at the 5′ end of the latter. (PDF 1357 kb) [file 12864_2018_5070_MOESM7_ESM.pdf]

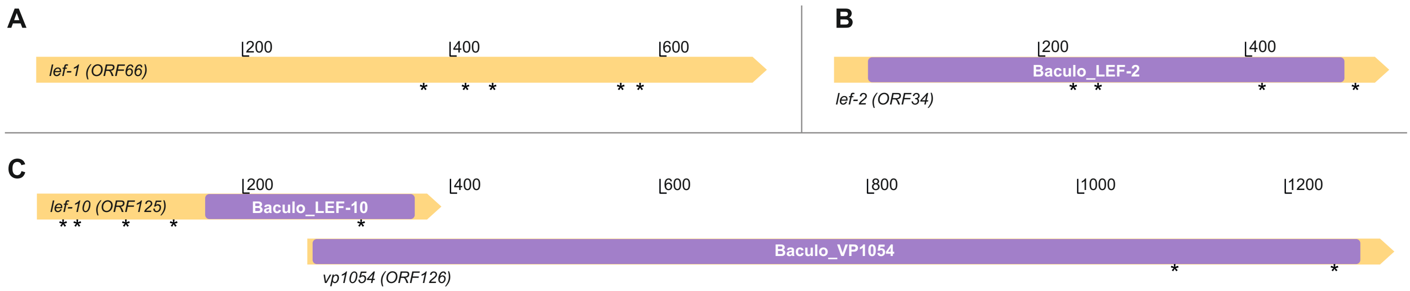

Additional File 5. Polymorphisms in *lef* genes. Non-synonymous mutations in proteins encoded by *lef-1* (A) and *lef-2* (B) were mainly found on their C-terminal. For LEF-10, polymorphisms were found mainly on it N-terminal (C). The exception is a single non-synonymous substitution at a region overlapping with vp1054, which in turn causes a synonymous change (CTG→CTA) at the 5' end of the latter.
