# Supplementary material for: Genome-wide diversity in temporal and regional populations of the betabaculovirus Erinnyis ello granulovirus (ErelGV)
Source: BMC Genomics. 2018 Sep 24;19:698. doi: 10.1186/s12864-018-5070-6 (PMC6154946; doi:10.1186/s12864-018-5070-6)
Supplement: Supplementary file 8 — Showing quantitative data on the genetic diversity of ErelGV isolates, and their consensus genome (combined diversity). Core genes are highlighted in bold. NSS = Number of non-synonimous substitutions. NSS/bp is shown as a factor of 10− 3. AUX = Auxiliary; MOD = Host modulation; REP = Replication; STR = Structural; TRA = Transcription; UNK = Unknown function. (PDF 1545 kb) [file 12864_2018_5070_MOESM8_ESM.pdf]

Additional File 8. Quantitative data on the genetic diversity of ErelGV isolates, and their consensus genome (combined diversity). Core genes are highlighted in **bold.** NSS = Number of non-synonimous substitutions. NSS/bp is shown as a factor of 10^{-3}. AUX = Auxiliary; MOD = Host modulation; REP = Replication; STR = Structural; TRA = Transcription; UNK = Unknown function.

ORF	Gene	Size (bp)	Function	ErelGV-1986		ErelGV-1994		ErelGV-1998		ErelGV-1999		ErelGV-2000		ErelGV-Acre		ErelGV-Pará		ErelGV (Sp.)	
				NSS	NSS/bp	NSS	NSS/bp	NSS	NSS/bp	NSS	NSS/bp	NSS	NSS/bp	NSS	NSS/bp	NSS	NSS/bp	NSS	NSS/bp
1	*granulin*	747	STR	0	0.00	0	0.00	0	0.00	0	0.00	0	0.00	0	0.00	0	0.00	0	0.00
2	*erel2*	339	UNK	0	0.00	0	0.00	0	0.00	0	0.00	2	5.90	0	0.00	0	0.00	2	5.90
3	*pk-1*	837	TRA	2	2.39	0	0.00	0	0.00	1	1.19	2	2.39	1	1.19	0	0.00	3	3.58
4	*erel4*	546	UNK	0	0.00	1	1.83	0	0.00	0	0.00	1	1.83	2	3.66	0	0.00	5	9.16
5	*dUTPase-like*	954	AUX	2	2.10	0	0.00	2	2.10	0	0.00	1	1.05	2	2.10	0	0.00	4	4.19
6	*erel6*	576	UNK	0	0.00	0	0.00	0	0.00	0	0.00	0	0.00	3	5.21	0	0.00	4	6.94
7	*erel7*	240	UNK	0	0.00	0	0.00	0	0.00	0	0.00	0	0.00	1	4.17	0	0.00	1	4.17
8	*ie-1*	1317	TRA	0	0.00	0	0.00	0	0.00	1	0.76	0	0.00	3	2.28	0	0.00	9	6.83
9	*erel9*	576	AUX	0	0.00	1	1.74	0	0.00	0	0.00	0	0.00	2	3.47	0	0.00	2	3.47
10	*erel10*	306	AUX	0	0.00	0	0.00	0	0.00	0	0.00	0	0.00	1	3.27	0	0.00	1	3.27
11	*erel11*	642	UNK	4	6.23	4	6.23	3	4.67	0	0.00	0	0.00	5	7.79	2	3.12	7	10.90
12	*erel12*	159	UNK	0	0.00	0	0.00	0	0.00	0	0.00	0	0.00	1	6.29	0	0.00	1	6.29
13	***odv-e18***	276	STR	0	0.00	0	0.00	0	0.00	0	0.00	0	0.00	0	0.00	0	0.00	0	0.00
14	***p49***	1380	STR	0	0.00	0	0.00	0	0.00	0	0.00	1	0.72	4	2.90	0	0.00	5	3.62
15	*erel15*	225	UNK	0	0.00	0	0.00	0	0.00	0	0.00	1	4.44	3	13.33	0	0.00	4	17.78
16	***pif-5/odv-e56***	1059	STR	1	0.94	0	0.00	0	0.00	0	0.00	1	0.94	3	2.83	0	0.00	3	2.83
17	*erel17*	480	STR	0	0.00	0	0.00	0	0.00	0	0.00	0	0.00	7	14.58	0	0.00	9	18.75
18	*erel18*	399	UNK	1	2.51	0	0.00	0	0.00	0	0.00	1	2.51	3	7.52	0	0.00	9	22.56
19	*erel19*	183	UNK	0	0.00	0	0.00	0	0.00	0	0.00	0	0.00	1	5.46	0	0.00	4	21.86
20	*pep-1*	528	STR	0	0.00	0	0.00	0	0.00	0	0.00	0	0.00	5	9.47	0	0.00	5	9.47
21	*pep/p10*	1026	STR	0	0.00	0	0.00	0	0.00	0	0.00	0	0.00	7	6.82	0	0.00	7	6.82
22	*pep-2*	453	STR	0	0.00	0	0.00	0	0.00	0	0.00	1	2.21	1	2.21	0	0.00	1	2.21
23	*erel23*	1143	AUX	2	1.75	1	0.87	2	1.75	0	0.00	1	0.87	7	6.12	0	0.00	7	6.12
24	*erel24*	162	UNK	1	6.17	1	6.17	1	6.17	1	6.17	2	12.35	1	6.17	0	0.00	3	18.52
25	*erel25*	285	UNK	1	3.51	0	0.00	1	3.51	0	0.00	1	3.51	1	3.51	0	0.00	2	7.02
26	*erel26*	1233	UNK	2	1.62	0	0.00	3	2.43	3	2.43	1	0.81	3	2.43	0	0.00	7	5.68
27	*erel27*	189	UNK	0	0.00	0	0.00	0	0.00	0	0.00	1	5.29	1	5.29	0	0.00	2	10.58
28	*f protein/efp*	1782	STR	6	3.37	0	0.00	5	2.81	5	2.81	4	2.24	6	3.37	0	0.00	8	4.49
29	*erel29*	930	UNK	5	5.38	0	0.00	5	5.38	5	5.38	1	1.08	6	6.45	0	0.00	16	17.20
30	*erel30*	711	UNK	4	5.63	0	0.00	6	8.44	7	9.85	1	1.41	1	1.41	0	0.00	8	11.25

ORF	Gene	Size (bp)	Function	ErelGV-1986		ErelGV-1994		ErelGV-1998		ErelGV-1999		ErelGV-2000		ErelGV-Acre		ErelGV-Pará		ErelGV (Sp.)	
				NSS	NSS/bp	NSS	NSS/bp	NSS	NSS/bp	NSS	NSS/bp	NSS	NSS/bp	NSS	NSS/bp	NSS	NSS/bp	NSS	NSS/bp
31	***pif-3***	567	STR	1	1.76	0	0.00	0	0.00	1	1.76	2	3.53	8	14.11	1	1.76	9	15.87
32	*erel32*	312	UNK	0	0.00	0	0.00	0	0.00	0	0.00	1	3.21	2	6.41	0	0.00	2	6.41
33	*erel33*	315	UNK	0	0.00	0	0.00	0	0.00	0	0.00	1	3.17	4	12.70	0	0.00	4	12.70
34	***lef-2***	528	TRA	0	0.00	0	0.00	2	3.79	2	3.79	3	5.68	3	5.68	0	0.00	3	5.68
35	*erel35*	273	UNK	0	0.00	0	0.00	1	3.66	0	0.00	1	3.66	0	0.00	0	0.00	1	3.66
36	*he65-like*	780	AUX	1	1.28	0	0.00	0	0.00	0	0.00	0	0.00	1	1.28	0	0.00	2	2.56
37	*erel37*	339	UNK	2	5.90	0	0.00	2	5.90	2	5.90	2	5.90	6	17.70	0	0.00	6	17.70
38	*erel38*	444	UNK	0	0.00	0	0.00	0	0.00	0	0.00	0	0.00	3	6.76	0	0.00	3	6.76
39	*mp-nase*	1389	AUX	0	0.00	0	0.00	0	0.00	0	0.00	1	0.72	3	2.16	0	0.00	5	3.60
40	*p13*	846	AUX	0	0.00	0	0.00	0	0.00	0	0.00	1	1.18	4	4.73	0	0.00	6	7.09
41	*chtBP*	258	AUX	0	0.00	0	0.00	0	0.00	0	0.00	1	3.88	1	3.88	0	0.00	1	3.88
42	***pif-2***	1125	STR	0	0.00	0	0.00	0	0.00	0	0.00	1	0.89	3	2.67	0	0.00	3	2.67
43	*pp-1*	291	AUX	1	3.44	0	0.00	0	0.00	0	0.00	0	0.00	0	0.00	0	0.00	2	6.87
44	*erel44*	2949	UNK	0	0.00	0	0.00	1	0.34	0	0.00	6	2.03	23	7.80	0	0.00	25	8.48
45	*erel45*	654	UNK	0	0.00	0	0.00	0	0.00	0	0.00	0	0.00	2	3.06	0	0.00	2	3.06
46	*erel46*	153	UNK	0	0.00	0	0.00	0	0.00	0	0.00	0	0.00	0	0.00	0	0.00	0	0.00
47	*v-ubq*	288	MOD	0	0.00	0	0.00	0	0.00	0	0.00	0	0.00	0	0.00	0	0.00	0	0.00
48	***odv-ec43***	1056	AUX	1	0.95	0	0.00	0	0.00	1	0.95	1	0.95	1	0.95	0	0.00	2	1.89
49	*erel49*	273	UNK	1	3.66	1	3.66	1	3.66	1	3.66	2	7.33	2	7.33	0	0.00	2	7.33
50	*39k/pp31*	837	TRA	1	1.19	1	1.19	1	1.19	0	0.00	0	0.00	1	1.19	0	0.00	1	1.19
51	*lef-11*	294	TRA	0	0.00	0	0.00	0	0.00	0	0.00	0	0.00	0	0.00	0	0.00	1	3.40
52	*sod*	474	AUX	1	2.11	1	2.11	1	2.11	1	2.11	1	2.11	1	2.11	1	2.11	2	4.22
53	*erel53*	192	UNK	2	10.42	2	10.42	2	10.42	2	10.42	3	15.63	3	15.63	0	0.00	3	15.63
54	*p10*	390	STR	2	5.13	1	2.56	3	7.69	0	0.00	0	0.00	6	15.38	0	0.00	21	53.85
55	***pif-0/p74***	1965	STR	6	3.05	3	1.53	6	3.05	1	0.51	4	2.04	7	3.56	1	0.51	12	6.11
56	*erel56*	378	UNK	2	5.29	2	5.29	2	5.29	2	5.29	3	7.94	2	5.29	0	0.00	4	10.58
57	*erel57*	720	UNK	1	1.39	1	1.39	1	1.39	1	1.39	2	2.78	4	5.56	0	0.00	4	5.56
58	*erel58*	606	UNK	0	0.00	0	0.00	0	0.00	0	0.00	0	0.00	3	4.95	0	0.00	3	4.95
59	*erel59*	174	UNK	0	0.00	0	0.00	1	5.75	0	0.00	0	0.00	4	22.99	0	0.00	5	28.74
60	*erel60*	270	UNK	0	0.00	0	0.00	0	0.00	0	0.00	0	0.00	1	3.70	0	0.00	1	3.70
61	***p47***	1182	TRA	0	0.00	0	0.00	0	0.00	0	0.00	1	0.85	11	9.31	0	0.00	11	9.31
62	*bv-e31*	666	AUX	0	0.00	0	0.00	0	0.00	0	0.00	0	0.00	0	0.00	0	0.00	1	1.50
63	*p24*	564	STR	0	0.00	0	0.00	0	0.00	0	0.00	0	0.00	1	1.77	0	0.00	1	1.77
64	*erel64*	288	UNK	0	0.00	0	0.00	0	0.00	0	0.00	1	3.47	1	3.47	0	0.00	2	6.94
65	*38.7k*	474	UNK	0	0.00	0	0.00	0	0.00	0	0.00	0	0.00	4	8.44	0	0.00	4	8.44

ORF	Gene	Size (bp)	Function	ErelGV-1986		ErelGV-1994		ErelGV-1998		ErelGV-1999		ErelGV-2000		ErelGV-Acre		ErelGV-Pará		ErelGV (Sp.)	
				NSS	NSS/bp	NSS	NSS/bp	NSS	NSS/bp	NSS	NSS/bp	NSS	NSS/bp	NSS	NSS/bp	NSS	NSS/bp	NSS	NSS/bp
66	***lef-1***	705	REP	0	0.00	0	0.00	0	0.00	0	0.00	2	2.84	5	7.09	0	0.00	5	7.09
67	***pif-1***	1611	STR	1	0.62	0	0.00	1	0.62	0	0.00	2	1.24	6	3.72	0	0.00	6	3.72
68	*fgf-1*	684	MOD	5	7.31	1	1.46	0	0.00	5	7.31	4	5.85	5	7.31	0	0.00	5	7.31
69	*erel69*	321	UNK	0	0.00	0	0.00	0	0.00	0	0.00	0	0.00	0	0.00	0	0.00	1	3.12
70	*erel70*	153	UNK	0	0.00	0	0.00	0	0.00	0	0.00	0	0.00	5	32.68	0	0.00	5	32.68
71	*erel71*	507	AUX	0	0.00	0	0.00	0	0.00	0	0.00	0	0.00	0	0.00	0	0.00	0	0.00
72	*lef-6*	303	TRA	0	0.00	0	0.00	1	3.30	0	0.00	0	0.00	0	0.00	0	0.00	1	3.30
73	*dbp*	846	REP	0	0.00	0	0.00	0	0.00	0	0.00	1	1.18	0	0.00	0	0.00	1	1.18
74	*erel74*	231	UNK	0	0.00	0	0.00	0	0.00	0	0.00	1	4.33	1	4.33	0	0.00	1	4.33
75	*erel75*	570	UNK	0	0.00	0	0.00	0	0.00	0	0.00	1	1.75	2	3.51	0	0.00	3	5.26
76	***p48***	1173	AUX	0	0.00	0	0.00	0	0.00	0	0.00	0	0.00	2	1.71	0	0.00	2	1.71
77	*erel77*	330	UNK	0	0.00	0	0.00	0	0.00	0	0.00	1	3.03	0	0.00	0	0.00	1	3.03
78	***erel78***	1125	STR	1	0.89	1	0.89	0	0.00	0	0.00	1	0.89	0	0.00	0	0.00	2	1.78
79	***p6.9***	180	STR	0	0.00	0	0.00	0	0.00	0	0.00	0	0.00	1	5.56	0	0.00	1	5.56
80	***lef-5***	729	TRA	1	1.37	0	0.00	0	0.00	1	1.37	0	0.00	1	1.37	0	0.00	1	1.37
81	***38K***	900	STR	1	1.11	0	0.00	0	0.00	2	2.22	1	1.11	2	2.22	0	0.00	4	4.44
82	***pif-4/odv-e28***	486	STR	2	4.12	0	0.00	2	4.12	2	4.12	2	4.12	0	0.00	0	0.00	2	4.12
83	***dna-helicase-1***	3303	REP	1	0.30	0	0.00	0	0.00	1	0.30	3	0.91	3	0.91	0	0.00	3	0.91
84	***odv-e25***	642	STR	1	1.56	2	3.12	2	3.12	1	1.56	1	1.56	2	3.12	0	0.00	2	3.12
85	***erel85***	489	AUX	2	4.09	0	0.00	3	6.13	2	4.09	2	4.09	2	4.09	0	0.00	3	6.13
86	***p33/sox***	765	MOD	1	1.31	0	0.00	1	1.31	1	1.31	1	1.31	2	2.61	0	0.00	2	2.61
87	***lef-4***	1368	TRA	2	1.46	0	0.00	2	1.46	2	1.46	3	2.19	6	4.39	0	0.00	8	5.85
88	***vp39capsid***	861	STR	0	0.00	0	0.00	0	0.00	0	0.00	0	0.00	1	1.16	0	0.00	1	1.16
89	***odv-e27***	855	STR	0	0.00	0	0.00	0	0.00	0	0.00	0	0.00	1	1.17	0	0.00	1	1.17
90	*erel90*	177	UNK	0	0.00	0	0.00	0	0.00	0	0.00	0	0.00	1	5.65	0	0.00	1	5.65
91	*erel91*	1101	UNK	1	0.91	1	0.91	1	0.91	2	1.82	3	2.72	9	8.17	0	0.00	9	8.17
92	*erel92*	375	UNK	0	0.00	0	0.00	0	0.00	0	0.00	0	0.00	2	5.33	0	0.00	2	5.33
93	***p95/vp91***	1791	STR	1	0.56	0	0.00	0	0.00	0	0.00	1	0.56	10	5.58	0	0.00	10	5.58
94	*erel94*	408	UNK	1	2.45	0	0.00	1	2.45	1	2.45	1	2.45	1	2.45	0	0.00	1	2.45
95	***erel95***	582	STR	0	0.00	0	0.00	0	0.00	0	0.00	0	0.00	0	0.00	0	0.00	0	0.00
96	***gp41***	837	STR	0	0.00	0	0.00	0	0.00	0	0.00	0	0.00	1	1.19	0	0.00	1	1.19
97	*iap-3*	810	MOD	0	0.00	0	0.00	0	0.00	0	0.00	0	0.00	3	3.70	0	0.00	7	8.64
98	***erel98***	291	UNK	0	0.00	0	0.00	0	0.00	0	0.00	0	0.00	0	0.00	0	0.00	0	0.00
99	***vlf-1***	1122	REP	0	0.00	0	0.00	0	0.00	0	0.00	1	0.89	0	0.00	0	0.00	1	0.89
100	*erel100*	669	UNK	0	0.00	0	0.00	0	0.00	0	0.00	1	1.49	8	11.96	0	0.00	8	11.96

ORF	Gene	Size (bp)	Function	ErelGV-1986		ErelGV-1994		ErelGV-1998		ErelGV-1999		ErelGV-2000		ErelGV-Acre		ErelGV-Pará		ErelGV (Sp.)	
				NSS	NSS/bp	NSS	NSS/bp	NSS	NSS/bp	NSS	NSS/bp	NSS	NSS/bp	NSS	NSS/bp	NSS	NSS/bp	NSS	NSS/bp
101	*erel101*	678	UNK	1	1.47	1	1.47	2	2.95	0	0.00	0	0.00	1	1.47	0	0.00	4	5.90
102	*erel102*	168	UNK	0	0.00	0	0.00	0	0.00	0	0.00	0	0.00	1	5.95	0	0.00	1	5.95
103	*erel103*	255	AUX	0	0.00	0	0.00	0	0.00	0	0.00	0	0.00	0	0.00	0	0.00	0	0.00
104	*erel104*	453	UNK	0	0.00	0	0.00	0	0.00	2	4.42	0	0.00	0	0.00	1	2.21	3	6.62
105	*p43-like*	1089	UNK	3	2.75	0	0.00	0	0.00	0	0.00	0	0.00	3	2.75	0	0.00	6	5.51
106	***dnapol***	3159	REP	4	1.27	1	0.32	3	0.95	0	0.00	2	0.63	4	1.27	0	0.00	10	3.17
107	***desmoplakin***	2094	STR	10	4.78	11	5.25	10	4.78	0	0.00	3	1.43	20	9.55	0	0.00	20	9.55
108	*lef-3*	1014	REP	0	0.00	0	0.00	0	0.00	0	0.00	0	0.00	1	0.99	0	0.00	1	0.99
109	***erel109***	378	STR	0	0.00	0	0.00	0	0.00	0	0.00	0	0.00	0	0.00	0	0.00	0	0.00
110	*erel110*	588	UNK	0	0.00	0	0.00	0	0.00	0	0.00	0	0.00	2	3.40	0	0.00	3	5.10
111	*iap-5*	843	MOD	0	0.00	0	0.00	0	0.00	0	0.00	0	0.00	2	2.37	0	0.00	2	2.37
112	***lef-9***	1482	TRA	1	0.67	0	0.00	0	0.00	0	0.00	1	0.67	4	2.70	0	0.00	4	2.70
113	*fp-25k*	465	AUX	0	0.00	0	0.00	0	0.00	0	0.00	1	2.15	1	2.15	1	2.15	2	4.30
114	*dna-ligase*	1674	REP	0	0.00	0	0.00	0	0.00	0	0.00	2	1.19	4	2.39	0	0.00	4	2.39
115	*erel115*	231	UNK	0	0.00	0	0.00	0	0.00	0	0.00	0	0.00	1	4.33	0	0.00	1	4.33
116	*erel116*	219	UNK	1	4.57	0	0.00	0	0.00	0	0.00	0	0.00	0	0.00	0	0.00	1	4.57
117	*fgf-2*	1197	MOD	1	0.84	0	0.00	1	0.84	2	1.67	4	3.34	4	3.34	0	0.00	4	3.34
118	*erel118*	279	UNK	0	0.00	0	0.00	0	0.00	0	0.00	0	0.00	1	3.58	0	0.00	1	3.58
119	***alk-exo***	1206	REP	0	0.00	0	0.00	0	0.00	0	0.00	1	0.83	2	1.66	0	0.00	3	2.49
120	*dna-helicase-2*	1371	REP	0	0.00	0	0.00	0	0.00	2	1.46	4	2.92	3	2.19	1	0.73	10	7.29
121	*erel121*	1059	UNK	1	0.94	1	0.94	4	3.78	1	0.94	0	0.00	7	6.61	0	0.00	28	26.44
122	***lef-8***	2610	REP	1	0.38	1	0.38	1	0.38	0	0.00	1	0.38	7	2.68	0	0.00	8	3.07
123	***erel123***	399	STR	0	0.00	0	0.00	0	0.00	0	0.00	0	0.00	2	5.01	0	0.00	6	15.04
124	*erel124*	795	UNK	0	0.00	0	0.00	0	0.00	0	0.00	1	1.26	6	7.55	0	0.00	19	23.90
125	*lef-10*	387	TRA	2	5.17	0	0.00	0	0.00	0	0.00	0	0.00	2	5.17	0	0.00	4	10.34
126	***vp1054***	1011	STR	0	0.00	0	0.00	0	0.00	0	0.00	0	0.00	4	3.96	1	0.99	4	3.96
127	*erel127*	174	UNK	0	0.00	0	0.00	0	0.00	0	0.00	0	0.00	2	11.49	0	0.00	2	11.49
128	*fgf-3*	882	MOD	2	2.27	1	1.13	1	1.13	1	1.13	2	2.27	3	3.40	0	0.00	4	4.54
129	*egt*	1401	MOD	0	0.00	0	0.00	1	0.71	0	0.00	0	0.00	5	3.57	0	0.00	8	5.71
130	*me53*	981	REP	4	4.08	4	4.08	4	4.08	5	5.10	7	7.14	4	4.08	0	0.00	7	7.14
