# Supplementary material for: Genome-wide diversity in temporal and regional populations of the betabaculovirus Erinnyis ello granulovirus (ErelGV)
Source: BMC Genomics. 2018 Sep 24;19:698. doi: 10.1186/s12864-018-5070-6 (PMC6154946; doi:10.1186/s12864-018-5070-6)
Supplement: Supplementary file 4 — Showing a phylogram of Betabaculovirus and ErelGV isolates. The phylogeny was inferred using concatenated amino acid sequences of baculovirus core genes. ErelGV isolates are highlighted in grey. The baculoviral genera Alphabaculovirus (α = AcMNPV); and Gammabaculovirus (γ = NeabNPV); Deltabaculovirus (δ = CuniNPV) were included as outgroups, and the latter was used as root. Bootstrap values are indicated for each interior branch. (PDF 1353 kb) [file 12864_2018_5070_MOESM4_ESM.pdf]

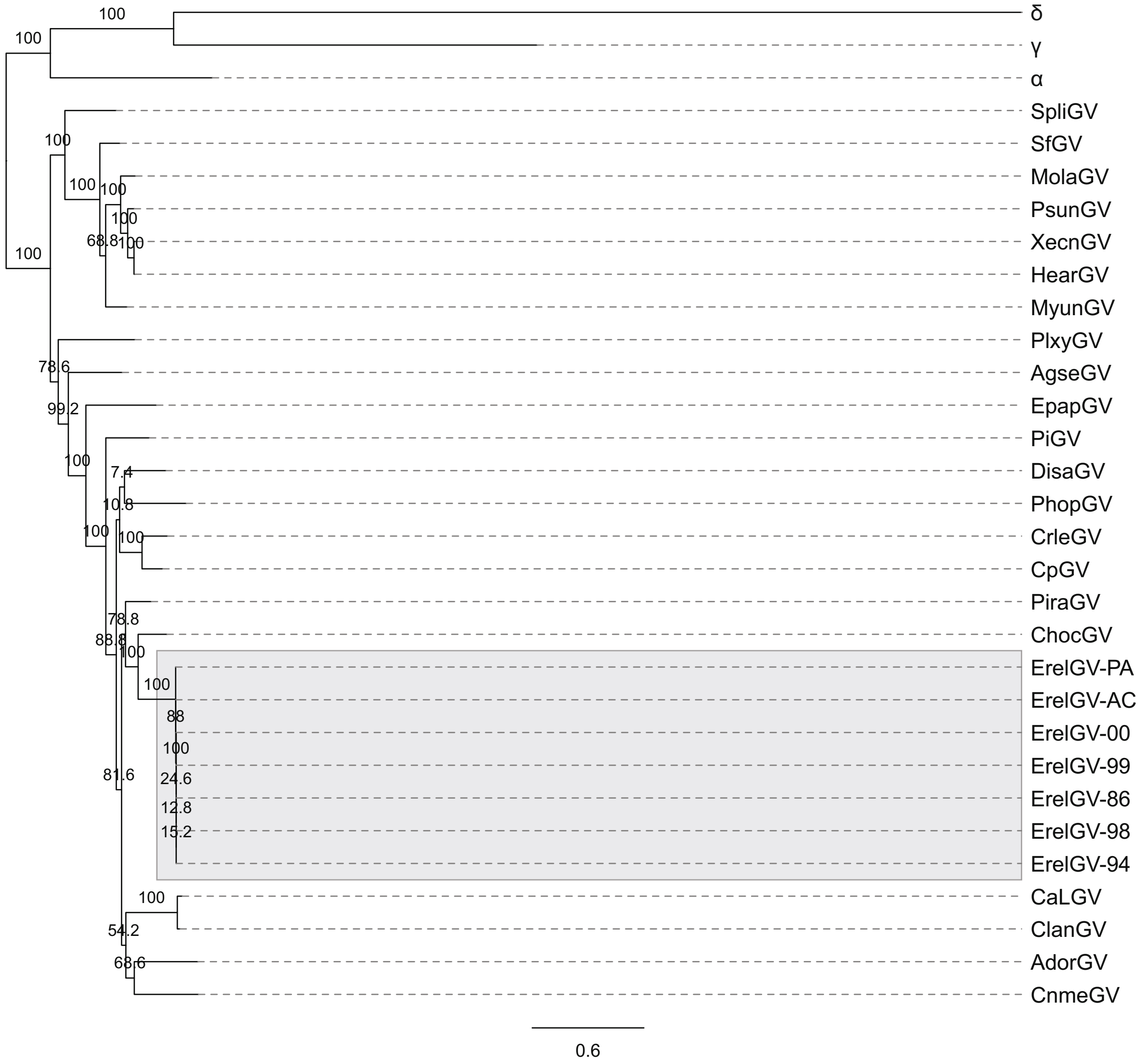

Additional File 2. Phylogram of Betabaculovirus and ErelGV isolates. The phylogeny was inferred using concatenated amino acid sequences of baculovirus core genes. ErelGV isolates are highlighted in grey. The baculoviral genera Alphabaculovirus (α = AcMNPV); and Gammabaculovirus (γ = NeabNPV); Deltabaculovirus (δ = CuniNPV) were included as outgroups, and the latter was used as root. Bootstrap values are indicated for each interior branch.
